# Cardiovascular Diseases Diagnosis Using an ECG Multi-Band Non-Linear Machine Learning Framework Analysis

**DOI:** 10.3390/bioengineering11010058

**Published:** 2024-01-07

**Authors:** Pedro Ribeiro, Joana Sá, Daniela Paiva, Pedro Miguel Rodrigues

**Affiliations:** CBQF—Centro de Biotecnologia e Química Fina, Laboratório Associado, Escola Superior de Biotecnologia, Universidade Católica Portuguesa, Rua de Diogo Botelho 1327, 4169-005 Porto, Portugal; s-pmsbribeiro@ucp.pt (P.R.); joanasa8@gmail.com (J.S.); danielaseabrapaiva@outlook.com (D.P.)

**Keywords:** ECG signals, cardiovascular diseases, machine learning models, discrete wavelet transform, non-linear analysis, discrimination

## Abstract

Background: cardiovascular diseases (CVDs), which encompass heart and blood vessel issues, stand as the leading cause of global mortality for many people. Methods: the present study intends to perform discrimination between seven well-known CVDs (bundle branch block, cardiomyopathy, myocarditis, myocardial hypertrophy, myocardial infarction, valvular heart disease, and dysrhythmia) and one healthy control group, respectively, by feeding a set of machine learning (ML) models with 10 non-linear features extracted every 1 s from electrocardiography (ECG) lead signals of a well-known ECG database (PTB diagnostic ECG database) using multi-band analysis performed by discrete wavelet transform (DWT). The ML models were trained and tested using a leave-one-out cross-validation approach, assessing the individual and combined capabilities of features, per each lead or combined, to distinguish between pairs of study groups and for conducting a comprehensive all vs. all analysis. Results: the Accuracy discrimination results ranged between 73% and 100%, the Recall between 68% and 100%, and the AUC between 0.42 and 1. Conclusions: the results suggest that our method is a good tool for distinguishing CVDs, offering significant advantages over other studies that used the same dataset, including a multi-class comparison group (all vs. all), a wider range of binary comparisons, and the use of classical non-linear analysis under ECG multi-band analysis performed by DWT.

## 1. Introduction

Heart and blood vessel problems, known as cardiovascular diseases (CVDs), are the main reason why many people die around the world [1]. According to the World Health Organization, 32% of global mortality is attributed to cardiovascular diseases, with the most prevalent being arrhythmias, cardiac arrests, and heart failure. It is estimated that CVDs take about 17.9 million lives every year [2]. Focusing on cardiac pathology and considering how much work the heart constantly does, it is amazing that it functions so well for a long time for many people. However, it can also experience problems and stop working properly due to risk factors like cholesterol, high blood pressure, cigarette smoking, diabetes mellitus, and adiposity [3].

Heart disease is a term for health issues that affect the heart’s function and condition. There are different types of heart disease, including: (1) Cardiomyopathy: heart muscle structural and functional abnormality without underlying coronary issues [4]; (2) Endocarditis: infection and inflammation of heart valves and inner lining [5]; (3) Myocarditis: inflammation of middle heart wall layer affecting blood pumping [6]; (4) Pericarditis: inflammation of the thin sac surrounding the heart [7]; (5) Coronary artery disease: cholesterol-filled plaques blocking heart arteries [8]; (6) Heart attack: sudden blockage of blood flow to heart muscle [9]; (7) Heart failure: symptoms include breathlessness, ankle swelling, and fatigue [10]; (8) Heart rhythm disorders (arrhythmias): irregular heartbeats [11]; (9) Sudden cardiac arrest: sudden stoppage of heartbeat [12]; (10) Heart valve disorders: issues with valves controlling blood flow [13]; (11) Congenital heart disease: heart abnormalities present from birth [14].

The beginning of the diagnosis of heart disease involves evaluating the patient’s medical history and conducting a physical examination. Afterwards, laboratory tests and/or additional non-invasive and invasive diagnostic exams can be performed [2]. Natriuretic peptides are the most common laboratory tests used to diagnose heart diseases. They can help identify individuals at higher risk of sudden cardiac death in the general population or patients with coronary artery disease [11]. However, several other non-invasive and invasive tests can be performed: (1) Electrocardiogram (ECG) and ambulatory monitoring: the 12-lead ECG is a key diagnostic test for cardiovascular diseases, assessing risk, and identifying arrhythmias [11]. Choose monitoring time based on symptom frequency. Holter for daily arrhythmias, patient-activated ECG for less frequent events, and ILRs for serious cases [11,15]; (2) Stress tests: monitor the heart during treadmill/bike exercise to assess response and detect exercise-related disorders like arrhythmias, ventricular tachycardia, coronary artery disease, and long QT syndrome [11,16]. Exercise tests aid in diagnosing long QT syndrome by measuring the QTc interval after 4 min of exercise [16]; (3) Imaging tests: essential for assessing heart function and detecting problems like cardiomyopathies [17]. Negative results may indicate primary electrical diseases [4]; (4) Electrophysiological study: exam to diagnose and guide treatment, involving measuring cardiac intervals, controlling electrical stimulation, and mapping heart structures. Effectiveness varies based on heart condition, presence of spontaneous ventricular tachycardia, medication use, and stimulation mode [18]; (5) Provocative diagnostic tests: use sodium channel blockers, adenosine, or epinephrine to detect syndromes. Use acetylcholine or ergonovine to assess coronary spasm as the cause of ventricular fibrillation [19]; (6) Genetic testing: next-gen sequencing made genetic testing accessible. Comprehensive gene panels reveal variations causing or modifying features in syndromes like Brugada, long QT, and hypertrophic and dilated cardiomyopathy [20]; (7) Cardiac catheterisation: catheter inserted into a blood vessel, and guided to the heart with X-ray images and dye to check for blockages [11].

In recent years, there has been a notable surge in computational power, driven by advanced hardware, parallel computing, cloud resources, and increased data accessibility. These developments have significantly enhanced the applications of machine learning (ML) in the diagnosis of CVDs [21]. The role of ML in CVDs is pivotal, as it harnesses data from medical tests to improve diagnostics and management, reducing human error, improving efficiency, and enhancing patient outcomes [22]. It contributes to early disease detection, precise risk assessment, advanced image analysis, predictive modelling, tailored treatment plans, remote patient monitoring, and expedited drug discovery [23]. However, ongoing research is essential to enhance this critical field further and save lives. Thus, for this study, our method will focus on ML-based ECG signal analysis approaches for discriminating CVDs, and thus we present in Table 1 the state of the art of this topic. The heart, operating as a non-linear system, manifests its electrical activity through the ECG signal [24]. The inherent non-linearity underscores the inadequacy of traditional linear analyses and standard clinical features in comprehensively capturing the intricate dynamics of the ECG signal. This complexity is further underscored by the challenges posed to deep learning tools, as their extraction of features may lack explainable understanding. Consequently, a deeper comprehension of how these tools reach and compute features becomes imperative for a more robust interpretation of ECG signals. Unlike prevailing state-of-the-art methods for the topic (Table 1), which have typically abstained from incorporating non-linear feature extraction in their methodology, our study aims to explore a non-linear approach to ECG analysis supported by classical ML tools. By doing so, we want to seek a more comprehensive understanding that embraces the inherent complexities of the heart’s electrical behaviour for improving CVDs diagnosis. For that, we defined three objectives for this study:

- Introduce the utilisation of 10 non-linear features (entropies—approximate, logarithmic, and Shannon, correlation dimension, detrended fluctuation analysis, energy, Higuchi fractal dimension, Hurst exponent, Katz fractal dimension, and Lyapunov exponent) extracted under discrete wavelet transform multi-band ECG signal analysis for characterising CVDs.
- Enhance the evaluation of distinguishing between various CVDs by accessing and comparing the individual and combined power of non-linear features.
- Evaluate the discriminatory performance of these features by inputting them into a comprehensive set of ML models.

The fulfilment of the goals will provide insights into the predictive power of these non-linear features, both independently and synergistically, contributing to a comprehensive understanding of their impact on CVD discrimination.

Finally, the paper is divided into five major sections in terms of structure. In Section 2, the applied methodology, including the database, signal processing, and feature extraction, is explained. The study results are indicated in Section 3 and discussed in Section 4. Finally, Section 5 draws the study conclusions.

## 2. Methodology

**Figure 1:**
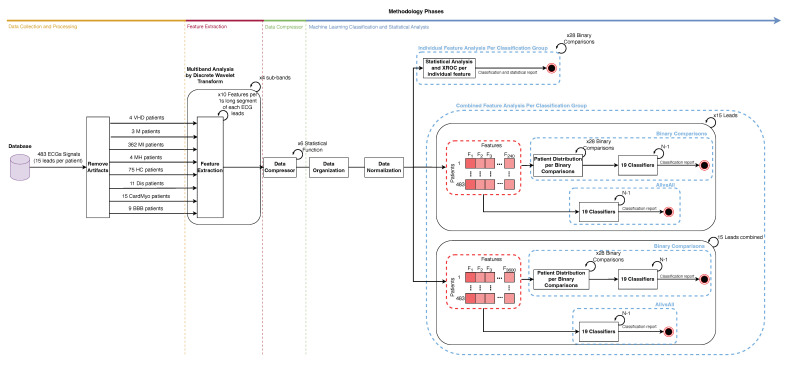
Workflow diagram.

This proposed methodology, illustrated in Figure 1, is split into 4 main parts:

- Data collection/pre-processing and artifacts removal;
- Feature extraction;
- Data compressor;
- Machine Learning classification and statistical analysis.

### 2.1. Experimental Setup

This study involved the use of two distinct programming languages: MATLAB and Python. MATLAB (version R2022a) was employed to eliminate noise from the ECG signal, extract non-linear features from the ECG data, and compress and structure the data for classification purposes. Python (version 3.9.12) was utilised to develop and implement various ML models and generate a discrimination report based on the obtained results. The choice between MATLAB and Python programming languages is driven by optimisation needs: MATLAB is particularly proficient in signal processing and feature extraction with highly optimised toolboxes, whereas Python takes the lead in optimising ML models.

This research was conducted using a MacBook Pro 14 equipped with an M1 Pro chip featuring an 8-core CPU, a 14-core GPU, and 16 GB of RAM.

### 2.2. Database Characterisation

The PTB diagnostic ECG database [43] comprises data from seven distinct cardiovascular disease groups as well as a healthy control group. This dataset consists of a total of 512 ECG records, each containing 12 conventional leads (I, II, III, αVr, αVl, αVf, V1, V2, V3, V4, V5, and V6), along with 3 ECG Frank leads (Vx, Vy, and Vz). The ECG data have been digitised at a sampling frequency of 1000 Hz.

Each lead contains ECG signal samples of 10 s and has been recorded by an electroencephalograph with: (1) input voltage: ±16 mV; (2) input resistance: 100 Ω (DC); (3) bandwidth: 0–1 kHz; and (4) noise voltage: 10 μV.

Table 2 represents the number of ECGs per diagnostic class present in the database.

### 2.3. Artifacts Removal

The ECG signals’ raw data in the database showed artifacts. To ensure the signal quality, complete signal deletion was performed. In the beginning, the database had 512 records. After the removal stage, the number of available signals in the database for the following tasks was reduced to 483 ECG records. Table 3 represents the number of ECGs per diagnostic class after the removal.

### 2.4. Signal Normalisation

The ECG signals, x(n), were loaded into MATLAB^®^ and normalised according to the following equation [44].

(1) $$x\left(n\right)=\frac{x(n)}{\sum_{n=0}^{N-1}x^{2}\left(n\right)},$$

where *N* represents the signal’s length. Then its mean value was removed.

#### Multi-Band Decomposition via Wavelet Transform and Features Extraction

The discrete-time wavelet transform (DWT) is a powerful technique used to analyse discrete-time signals with finite energy. It involves breaking down the signal into a set of basis functions composed of a limited number of prototype sequences and their time-shifted variations. This process, as described in Guido’s research in 2022 [45], offers significant advantages for analysing signals in the time–frequency domain. By seamlessly transitioning between the time and frequency domains, it enables the localisation of the source of frequency compounds in time.

To perform the decomposition and subsequent reconstruction, an octave-band critically decimated filter bank is employed. This approach, pioneered by Malvar in 1992 and further developed by Vetterli in 1995 [46,47], provides an effective framework. When considering only the positive frequencies, each sub-band in the transform is confined to a specific range,

(2) $$W_{k}=\left\{\begin{array}{cc} \left[0,\pi /2^{S}\right], & m=0,\\ \left[\pi /2^{S-m+1},\pi /2^{S-m}\right], & m=1,2,\ldots ,S, \end{array}\right.$$

where *S* is the number of levels, S+1 is the number of sub-bands, and π is the normalised angular frequency equivalent to half the sampling rate.

The DWT employs an analysis scale function, denoted as ${\tilde{\phi }}_{1}\left(n\right)$, and an analysis wavelet function, denoted as ${\tilde{\psi }}_{1}\left(n\right)$, which are defined as follows:

(3) $${\tilde{\phi }}_{1}\left(n\right)=h_{\mathrm{LP}}\left(n\right)$$

and

(4) $${\tilde{\psi }}_{1}\left(n\right)=h_{\mathrm{HP}}\left(n\right),$$

where $h_{\mathrm{LP}}\left(n\right)$ and $h_{\mathrm{HP}}\left(n\right)$ represent the impulse responses of the analysis filters for the half-band low-pass and high-pass components, respectively.

Defining the following recursion formulas

(5) $$\begin{array}{c} {\tilde{\phi }}_{i+1}\left(n\right)={\tilde{\phi }}_{i}\left(n/2\right)*{\tilde{\phi }}_{1}\left(n\right), \end{array}$$

(6) $$\begin{array}{c} {\tilde{\psi }}_{i+1}\left(n\right)={\tilde{\phi }}_{i}\left(n\right)*{\tilde{\psi }}_{1}\left(n/2^{i}\right), \end{array}$$

where the symbol “∗” signifies the convolution operation, the analysis filter corresponding to the *m*th sub-band is expressed as follows:

(7) $$h_{m}\left(n\right)=\left\{\begin{array}{cc} {\tilde{\phi }}_{S}\left(n\right), & m=0,\\ {\tilde{\psi }}_{S+1-m}\left(n\right), & m=1,2,\ldots ,S. \end{array}\right.$$

The *m*th sub-band signal is computed as

(8) $$x_{m}\left(n\right)=\left\{\begin{array}{cc} \sum_{k=-\infty }^{\infty }x\left(k\right)h_{m}\left(2^{S}n-k\right), & m=0,\\ \sum_{k=-\infty }^{\infty }x\left(k\right)h_{m}\left(2^{S-m+1}n-k\right), & m=1,2,\ldots ,S. \end{array}\right.$$

In this research, the DWT was employed to decompose each ECG segment of 1 s length into sub-bands ($x_{m}\left(n\right)$) up to level three (S=3). The applied wavelet was Symlet7, and this wavelet proved to be good for ECG signals analysis until decomposition at level 3 [48,49]. To ensure consistency with the original sampling rate, the sub-band signals, $x_{m}\left(n\right)$, underwent re-sampling using the wavelet interpolation method [50]. After that, 10 non-linear features (check Table 4 for more information) were collected from each signal sub-band of 1 s length from a total of 10 s signal length. Then, the resulting time series per feature and sub-band were compressed over time, respectively, by 6 distinct statistical functions: average (Avg), standard deviation (Std), 95th percentile (P95), variance (Var), median (Med), and kurtosis (Kur) [49]. At the end of the process, the data matrix, comprised of all 10-second time series vectors of features extracted from all sub-bands over time for all patients, underwent normalisation using the z-score method [51].

### 2.5. Data Driven Framework Analysis

#### 2.5.1. Individual Feature Power Analysis over Binary Groups

The evaluation of the discriminating power of each feature distribution between pairs of study groups, such as VHDvs.M, VHDvs.MI, VHDvs.MH, VHDvs.HC, VHDvs.Dis, VHDvs.CardMyo, VHDvs.BBB, Mvs.MI, Mvs.MH, Mvs.HC, Mvs.Dis, Mvs.CardMyo, Mvs.BBB, MIvs.MH, MIvs.HC, MIvs.Dis, MIvs.CardMyo, MIvs.BBB, MHvs.HC, MHvs.Dis, MHvs.CardMyo, MHvs.BBB, HCvs.Dis, HCvs.CardMyo, and HCvs.BBB, was conducted using the XROC classifier [58], a binary classifier working within a leave-one-out cross-validation process, and using the Mann–Whitney test. A total of 3600 features, consisting of 10 non-linear features time series compressed over time by (×) 6 statistical measures over (×) 4 sub-bands for each one of the (×) 15 leads per participant, were individually assessed to measure their potential to differentiate between these groups. The methodology variation to perform individual feature assessment for discrimination is signalised in Figure 1. It should be noted that the normality and homoscedasticity of each one of the time series feature vector distributions have been assessed for distinguishing binary classes with the MATLAB function ktest, which performs the Kolmogorov–Smirnov and Levene tests, respectively. The hypothesis of parametric tests was not met, so we applied a non-parametric test, such as the Mann–Whitney test.

#### 2.5.2. Combined Features Power Analysis for Groups Discrimination Using Sci-Learn ML Models

In this case, the model’s performance for discriminating between pairs of study groups and between Allvs.All was evaluated by feeding 19 selected Sci-learn ML models [59], presented in Table 5, with combined features—240 features (10 features extracted from (×) 4 sub-bands and compressed (×) by 6 statistics) for the individual lead case or 3600 features (240 features per lead × 15 leads) per combined leads case, for each group comparison, within a leave-one-out cross-validation procedure. The methodology variation to perform combined feature assessment for discrimination is signalised in Figure 1.

#### 2.5.3. Classification Metrics

The model’s performance evaluation was carried out using 9 metrics: Accuracy, Precision, Recall, F1-Score, AUC, Kappa, MCC, CSI, and Gmean.

The Accuracy represents the number of corrected classified classes concerning all cases [60] and can be defined as

(9) $$Accuracy=\frac{TP+TN}{TP+TN+FP+FN}\times 100\%,$$

where, a TP, TN, FP, and FN are, respectively, the true positives, true negatives, false positives, and false negatives [61].

The Precision, also known as a positive predictive value, shows the proportion of well-classified positive cases to the total cases predicted as positive [62]. The Precision can be defined as

(10) $$Precision=\frac{TP}{TP+FP}\times 100\%.$$

The Recall, defined as

(11) $$Recall=\frac{TP}{TP+FN}\times 100\%,$$

represents the proportion of correctly predicted positive cases concerning the total number of positive cases [62].

The F1-Score is the harmonic average between the Recall and the *Precision* [63], and the equation is defined as

(12) $$F1\text{-}score=\frac{2\times Precision\times Recall}{Precision+Recall}\times 100\%.$$

The Kappa normalises the Accuracy by the possibility of agreement by chance [64] and is defined as

(13) $$Kappa=\frac{2\times (TP\times TN-FN\times FP)}{\left(TP+FP)\times (FP+TN)+(TP+FN)\times (FN+TN\right)}.$$

The MCC is useful for uneven data [65]. It varies between 0 and 1, with 0 as the worst scenario and 1 as the best. It is defined as

(14) $$MCC=\frac{TP\times TN-FP\times FN}{\sqrt{\left(TP+FP)\times (TP+FN)\times (TN+FP)\times (TN+FN\right)}}.$$

The CSI provides a more nuanced evaluation of a binary classification model’s effectiveness by considering both the correct identification of positive instances and the ability to avoid false positives. [66]. The CSI equation can be defined as

(15) $$CSI=\frac{TP}{TP+FP+FN}.$$

The Gmean is the measure that considers a balance between the performance of all classes. The higher the value is, the lower is the risk of models over-fitting. It is defined as

(16) $$Gmean=\sqrt{Recall\times Specificity},$$

where Specificity is defined as

(17) $$Specificity=\frac{TN}{FN+TN}.$$

The area under the curve (AUC) of the receiver operating characteristic curve (ROC) is a metric that evaluates how well a model can distinguish between positive and negative classes. It achieves this by comparing the rate of TP against the rate of FP at different classification thresholds. The value of AUC ranges between 0 and 1, with the perfect classifier resulting in a value of 1, while a random classifier has an AUC of 0.5. Using AUC allows for a single-value measure of a model’s performance. This is especially useful for comparing models and assessing performance in scenarios where there is an imbalance between classes [67].

## 3. Results

Table 6 displays the individual features’ discrimination power that yielded the best results for statistical and XROC analysis conducted across all 28 binary comparisons (VHDvs.M, VHDvs.MI, VHDvs.MH, VHDvs.HC, VHDvs.Dis, VHDvs.CardMyo, VHDvs.BBB, Mvs.MI, Mvs.MH, Mvs.HC, Mvs.Dis, Mvs.CardMyo, Mvs.BBB, MIvs.MH, MIvs.HC, MIvs.Dis, MIvs.CardMyo, MIvs.BBB, MHvs.HC, MHvs.Dis, MHvs.CardMyo, MHvs.BBB, HCvs.Dis, HCvs.CardMyo, HCvs.BBB, Disvs.CardMyo, Disvs.BBB, and CardMyovs.BBB), respectively.

Table 7 shows the number of occasions where a feature distribution is shown to be significant (p<0.05) for separating binary classes.

**Figure 2:**
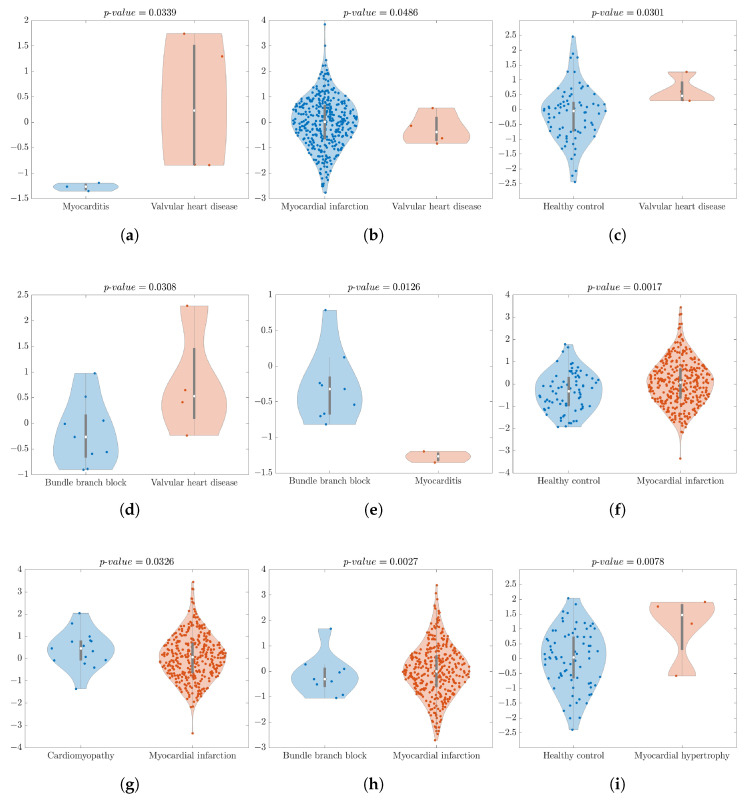

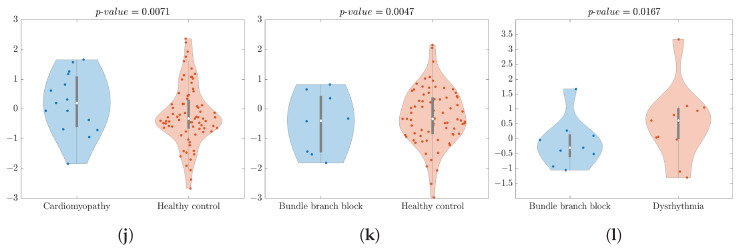
Violin plots of binary group distributions with significant differences—individual feature power analysis for discrimination. (**a**) VHDvs.M; (**b**) VHDvs.MI; (**c**) VHDvs.HC; (**d**) VHDvs.BBB; (**e**) Mvs.BBB; (**f**) MIvs.HC; (**g**) MIvs.CardMyo; (**h**) MIvs.BBB; (**i**) MHvs.HC; (**j**) HCvs.CardMyo; (**k**) HCvs.BBB; (**l**) Disvs.BBB.

Figure 2 illustrates the violin plots for the comparison groups where there was a significant difference, reported in Table 6.

**Figure 3:**
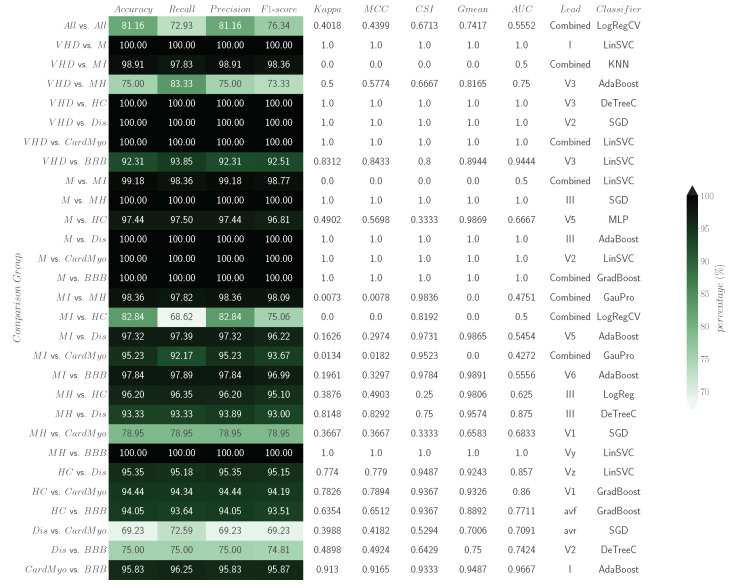
Heatmap classification report regarding combined feature discriminant power analysis—the best Accuracy, Recall, Precision, *F*1-Score, AUC, Kappa, MCC, CSI, and Gmean results for each comparison group plus the information of lead and ML classifier applied for signal analysis.

The classification results regarding combined feature power analysis performed by ML classifiers can be found as a heatmap in Figure 3.

**Figure 4:**
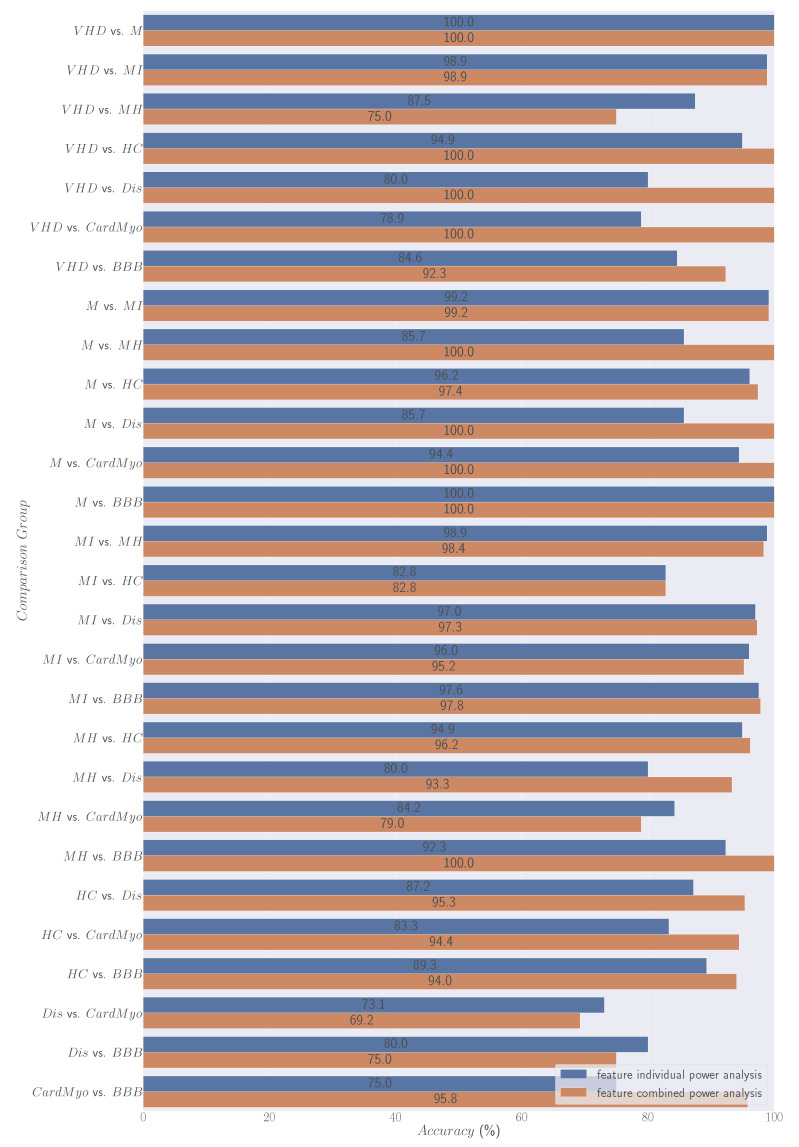
Direct comparison using Accuracy between individual and combined feature power analyses for binary groups’ discrimination performed by ML models.

The direct comparison between the individual and combined feature power analyses for discrimination is shown in Figure 4.

## 4. Discussion

For a more comprehensive discussion, we divided this section into three subsections according to the two variations of analysis employed in this study—individual and combined feature power analyses for discrimination—and compared our results with state-of-the-art results. While acknowledging that, in medicine, Accuracy may not fully capture the balance between Recall and Specificity, our discussion will primarily focus on Accuracy for checking the model’s performance as it enables a more direct comparison of our results with those achieved by state-of-the-art methods.

### 4.1. Data Driven Analysis—Individual Feature Power Analysis

From a broader perspective, we can observe in Table 6 that the top-performing feature, compressor, and wavelet sub-band were the CorrDim, Avg, and 2nd sub-band (DWT details 2nd level), respectively. Notably, they were present in 100% of the best results for comparison groups, encompassing all 28 binary comparison groups.

In addition, 12 of the 28 binary comparisons were shown to be statistically significant (42.86% of all analyses) and, out of the 15 leads utilised in this study, 8 exhibited at least one analysis with statistically significant differences. The most frequently represented lead in the table was V2, appearing in 16% of the cases (4 out of 28 binary groups).

The classes most frequently represented in binary groups with significant differences were the HC and the BBB classes. Both classes were present in 5 out of the 12 comparison groups with significant differences (41.67% of the cases). Additionally, the CardMyo and MH classes were the only ones that did not show significant differences when compared with the BBB class, and the *M* and Dis classes did not show significant differences when compared with the HC class.

Regarding each binary analysis:

- VHDvs.M analysis yielded a significant *p*-value of 0.0339, and an Accuracy and Recall of 100%. The feature CorrDim, with the compressor Avg, and lead V4 extracted from the 2nd sub-band provided excellent results. As shown in Figure 2a, the violin plot easily illustrates a distinct separation between these two classes.
- VHDvs.MI statistical analysis produced a significant result (*p*-value = 0.0486). The XROC analysis achieved an Accuracy of 98.91% and Recall of 0% for this binary comparison. The achieved Recall of 0% underscores one of the primary limitations of the dataset, namely its imbalance with the XROC, over-adjusting itself too much to the predominant class—MI, and it corroborates also the difficulty of splitting groups by the statistical test. In Figure 2b, we can see some outlier values but the highest density of the data is located close to the median.
- VHDvs.MH statistical analysis revealed a non-significance *p*-value. The XROC metrics—Accuracy and Recall—demonstrated strong performance for discrimination between groups, with values of 87.50% and 75.00%, respectively.
- VHDvs.HC group analysis displayed a significant difference, with a *p*-value of 0.0301. It achieved an Accuracy of 94.94% and a Recall of 0%. Figure 2c indicates some outliers in the HC class, but the highest data density is close to the median. Despite the good statistical analysis results, once more the XROC over-adjusts itself too much to the predominant class—HC, achieving a Recall of 0% for discriminating the class VHD. The imbalanced database and the HC’s large number of outliers contribute to these results. The XROC employs an averaging method within its genesis, which assigns significant weight to outliers in the final results.
- VHDvs.Dis analysis resulted in a non-significant *p*-value, an Accuracy of 80.00%, and a Recall of 100%. Despite being statistically non-significant, the XROC results showed a good performance for discriminating.
- VHDvs.CardMyo analysis resulted in a non-significant *p*-value. The XROC metrics Accuracy and Recall were 78.94% and 50.00%, respectively.
- VHDvs.BBB statistical analysis yielded a *p*-value of 0.0308, and the XROC achieved an Accuracy of 84.62% and Recall of 75.00%. Figure 2d illustrates a higher density of BBB’s data being located close to the median.
- Mvs.MI statistical analysis yielded a *p*-value of non-significance. The Accuracy achieved a value of 99.18% and the Recall resulted in 0%.
- Mvs.MH statistical analysis exhibited no significant difference in the *p*-value. The Accuracy and Recall reached 85.71% and 100%, respectively.
- Mvs.HC analysis also showed no significant *p*-value. Accuracy and Recall demonstrated an interesting performance, achieving values of 96.15% and 0%, respectively. This underscores the difficulty of the XROC classifier in accurately discriminating between unbalanced data sample sizes.
- Mvs.Dis analysis also revealed a non-significant *p*-value, with the Accuracy and Recall showcasing the values of 85.71% and 33.33%, respectively.
- Mvs.CardMyo analysis resulted in a non-significant *p*-value, an Accuracy of 94.44% and a Recall of 66.67%. Despite being statistically non-significant, the XROC showed good behaviour for discriminating between classes.
- Mvs.BBB statistical analysis indicated significant differences with a *p*-value of 0.0126. The Accuracy and Recall reached 100%. In Figure 2e, the violin plot displayed an outlier in the BBB class, but the highest data density was slightly below the median. It is worth noting that there was a clear separation between the two classes.
- MIvs.MH analysis resulted in a non-significant *p*-value. Despite that, the XROC performed well with a discrimination Accuracy of 98.91% and a Recall of 100%.
- MIvs.HC statistical analysis yielded a *p*-value of 0.0017. The Accuracy and the Recall achieved values of 82.84% and 100%, respectively. In Figure 2f, the violin plot exhibited some outliers, but the highest data density was close to the median for both classes.
- MIvs.Dis analysis was shown to be non-significant. The XROC metrics of Accuracy and Recall displayed significant performance percentages, with values of 97.05% and 100%, respectively.
- MIvs.CardMyo statistical analysis revealed a *p*-value of 0.0326 alongside impressive classification metrics, boasting an Accuracy of 96.29% and a perfect Recall of 100%. In Figure 2g, the violin plot exhibited some outliers, but the highest data density was close to the median for both classes.
- MIvs.BBB analysis showed statistical significance and the Accuracy stood at 97.57%, with a flawless Recall of 100%. Figure 2h gives us the opportunity to see some outliers in both classes but the majority of the data were located close to the median.
- MHvs.HC analysis achieved a significant *p*-value, reaching a statistical analysis value of 0.0078. The Accuracy was 94.94% and the Recall was 0%, which perfectly illustrates the imbalance of the dataset. Figure 2i shows the HC class, with the highest density of data close to the median.
- MHvs.Dis analysis exhibited non-significant *p*-values, with Accuracy rates of 80.00% and 50.00%, respectively.
- MHvs.CardMyo demonstrated an Accuracy of 84.21% and a Recall of 50.00%, while the MHvs.BBB group yielded an Accuracy of 92.31% and a Recall of 75.00%, with both analyses showing a non-statistical significance. While statistical significance may be elusive, the consistently high Accuracy and Recall values underscore the potential efficacy of the model in discriminating between different conditions within the studied groups.
- HCvs.Dis analysis showed non-significant difference. The Accuracy and Recall displayed great performance, with values of 87.21% and 100%, respectively.
- HCvs.CardMyo showed a significant difference, with a *p*-value of 0.0071. The Accuracy and Recall exhibited strong performance, with values of 83.33% and 94.67%, respectively. In Figure 2j, the violin plot displayed some outliers in the HC class, but the highest data density was close to the median.
- HCvs.BBB analysis provided a significant *p*-value of 0.0047 accompanied by an Accuracy of 89.29% and an impressive Recall of 100%. Figure 2k shows the violin plot with some outliers in the HC class, but there was a higher density of data close to the median.
- Disvs.CardMyo comparison analysis yielded a non-significant *p*-value, with an Accuracy of 73.08% and a Recall of 54.54%.
- Disvs.BBB comparison analysis showed a *p*-value of 0.0167, achieving an Accuracy of 80.00% and a Recall of 81.81%. Figure 2l shows a violin plot with a couple of outliers in both classes, but the highest density of data was close to the median.
- CardMyovs.BBB analysis provided a non-significant *p*-value, with an Accuracy of 75.00% and a Recall of 73.33%.

Looking to Table 7, we can see the total number of occasions that a feature was demonstrated to be significant over binary groups and in total. It should be noted that each originally defined feature generated 360 features per analysis; for more information check Section 2.5.1. While CorrDim emerged as a standout performer individually, the results emphasise that the other nine features also demonstrated statistical significance in distinguishing between classes with more moments of appearing to be significant than actually CorrDim (523 vs. 600). MIvs.HC and HCvs.BBB showed the highest number of results with significant differences, which were 1185 and 1184, respectively. VHDvs.MI, VHDvs.BBB, Mvs.BBB, MHvs.HC, and Disvs.BBB were the binary groups with the lowest amount of occasions of significant feature distributions, 237 each.

### 4.2. Data Driven Analysis—Combined Feature Power Analysis

Figure 3 presents the classification metrics report for the comparison groups provided by 19 Sci-learn ML classifiers with combined features as entries. The heatmap employs a gradient of green shades in its colour scheme, serving to vividly illustrate the method’s discrimination capabilities for Accuracy, Recall, Precision, F1-Score, AUC, Kappa, MCC, CSI, and Gmean in each comparative analysis. Lighter shades of green represent lower discriminatory power, while deeper, richer greens signify higher effectiveness. By looking into the results, it can be seen that VHDvs.M, VHDvs.MI, VHDvs.HC, VHDvs.Dis, VHDvs.CardMyo, Mvs.MH, Mvs.Dis, Mvs.CardMyo, Mvs.BBB, and MHvs.BBB obtained 100% on all evaluation metrics. Comparing the individual power discrimination results presented in Table 6, it can be seen that generally the discrimination results have increased, and the ratio of 100% on all evaluated metrics per binary analysis has increased (2/28 to 10/28). Comparing the Accuracy results achieved through combined feature power analysis with those obtained through individual feature power analysis (see Figure 4 for a visual representation of the analysis for each binary comparison; this figure provides a clear and concise overview, facilitating an easy assessment of performance differences between the two approaches described in Section 2.5.1 and Section 2.5.2), we observe a significant overall improvement across all binary comparisons. Among the 28 comparisons conducted, the results indicate that 17 exhibits enhanced discrimination Accuracy when utilising combined features analysis. In contrast, in five instances, the Accuracy remained the same as that observed in individual feature power analysis. There are only six cases where we notice a decrease in Accuracy compared with individual power analysis (VHDvs.MH, MIvs.MH, MIvs.CardMyo, MHvs.CardMyo, Disvs.CardMyo, and Disvs.BBB).

Returning to the analysis of Figure 3, in Allvs.All, an Accuracy of 81.16%, Recall of 72.93%, Precision of 81.16%, 76.34% for the F1-Score, Kappa of 0.4018, MCC of 0.4399, CSI of 0.6713, Gmean of 0.7417, and AUC of 0.5552 were achieved. The leads ensemble combination was the most represented in the table, corresponding to 28% of the total appearances. The classifier with the most frequent appearances was LinSVC, representing 24% of the cases. The binary groups VHDvs.MH, MIvs.HC, MHvs.CardMyo, Disvs.CardMyo, and Disvs.BBB, exhibited Precision values below 90%. This challenge in correct classifying can be attributed to the close relationship between CardMyo and MH or Dis. In a clinical context, it is common for patients to present with CardMyo alongside either VHD or Dis [68,69]. This clinical overlap makes accurate differentiation challenging. Understanding and addressing these interconnected conditions are essential for improving classification Accuracy in these scenarios. The MIvs.HC classification, with an 82.84% Precision, presents challenges due to the potential presence of acute MI within the HC class. Additionally, some patients who have recovered from MI may be categorised as HC [70]. These factors contribute to a slightly lower classification performance of ML models for discrimination within this context.

Moreover, upon assessing various models and their performance metrics, a notable observation is the impact of the imbalanced dataset, particularly evident in comparison groups involving one of either MI or HC classes. In such instances, we observed a range of AUC results from 0.4272 to 0.6667 across all nine comparison groups where at least one of these two classes was present. These findings underscore the substantial challenge of distinguishing between unevenly represented classes. The Gmean further highlights the noteworthy observation that in 71.42% of cases (five out of seven binary comparisons) where the MI class is pitted against another class, the Gmean metric yields a result of 0. However, in comparisons involving MI against Dis and BBB, a lower risk of over-fitting is evident, with Gmean values of 0.9865 and 0.9891, respectively. The CSI metric reveals that the preponderance of comparison groups, specifically 17 out of 29, exhibits results surpassing 0.9. This observation underscores a notable challenge in classification, particularly when dealing with classes characterised by higher data abundance. The MCC metric highlights a notable trend, with 31% of the comparison groups (9 out of 29 groups) achieving perfect predictions, each obtaining a maximum value of 1. Notably, the class MI demonstrates the least favourable outcomes, with its highest MCC value capped at 0.3297 when included in a comparison group. The Kappa metric reveals a noteworthy pattern, with 31% of the comparison groups (9 out of 29 groups) achieving perfect agreement, each attaining a maximum value of 1. Additionally, 41.37% of the groups surpass a Kappa value higher than 0.083.

### 4.3. Study Results vs. State-of-the-Art Results

When we analyse Table 1, it becomes evident that our results closely match or slightly surpass the achievements of the state of the art, offering valuable insights for enhancing robustness. In particular, when considering the eight state-of-the-art studies that utilised the PTB database, our results are lower in the binary comparisons of MIvs.HC and HCvs.CardMyo, with differences of less than 13% and 0.76%, respectively. Furthermore, the present study offers significant advantages over other studies as it includes a multi-class comparison group (Allvs.All), a higher variety of binary comparisons, and the application of classical non-linear analysis under ECG multi-band analysis performed by DWT. These particularities allow a high capacity of differentiation of each class present in the database, a level of detail not typically found in state-of-the-art articles. Moreover, it is imperative to underscore that the developed algorithm relies on ECG signals, presenting distinctive advantages when compared with alternative diagnostic sources such as stress tests, imaging tests, electrophysiological studies, provocative diagnostic tests, genetic tests, and cardiac catheterisation, among others. The affordability, non-invasiveness, widespread use in clinical settings, and user-friendly nature of ECG make it an optimal choice. Its efficacy not only facilitates the easy adoption of our algorithm globally but also addresses the unique needs of patients unable to leave their hospital beds. This highlights the algorithm’s versatility and accessibility in diverse healthcare settings.

## 5. Conclusions

For this research, 10 non-linear features (En, ApEn, LogEn, ShaEn, EH, Elay, *H*, *K*, CorrDim, and DFA) were extracted from a well-known ECG database (PTB diagnostic ECG database). From the recorded 15 leads per patient (12 conventional leads and 3 Frank leads), each signal lead underwent a 1-second length non-overlapped windowing process over time for extracting a total of 10 non-linear features per window. At the end of the process, each feature time series was compacted by six statistics. The individual power and combined power were accessed from discriminating between different cardiovascular pathologies (VHDvs.M, VHDvs.MI, VHDvs.MH, VHDvs.HC, VHDvs.Dis, VHDvs.CardMyo, VHDvs.BBB, Mvs.MI, Mvs.MH, Mvs.HC, Mvs.Dis, Mvs.CardMyo, Mvs.BBB, MIvs.MH, MIvs.HC, MIvs.Dis, MIvs.CardMyo, MIvs.BBB, MHvs.HC, MHvs.Dis, MHvs.CardMyo, MHvs.BBB, HCvs.Dis, HCvs.CardMyo and HCvs.BBB), Disvs.CardMyo, Disvs.BBB), and CardMyovs.BBB) and one multi-class comparison (Allvs.All).

The Accuracy discrimination results ranged between 81% and 100%. The results demonstrate that the applied method serves as a robust tool for effectively distinguishing cardiovascular diseases (CVDs) through the analysis of ECG signals. The level of detail and discrimination achieved surpasses what is typically observed in state-of-the-art studies using the same dataset. Despite our results indicating a great ability of the proposed method to diagnose, offering in this way another alternative avenue for medical doctors to arrive at more confident diagnoses, this study had some limitations. (1) The inherently technical nature of utilising unusual standard clinical features extracted from ECG signals may hinder complete interpretability from a clinician’s standpoint. This could pose a challenge to its rapid and widespread integration into clinical practice. (2) The high computation time of multi-band analysis for the chosen methodology led us to choose just one wavelet (Symlet7) from tens of wavelets with the level of decomposition set to 3, based on prior work [48,49], as the main wavelet. A more meticulous analysis needs to be carried out in future to choose the wavelet and level of decomposition that adjusts itself better to each CVD activity. (3) The results should be further enhanced by updating them with a larger and more balanced population to ensure a more reliable generalisation and to split data as hold-out for classifying (e.g., 70% for training and 30% for testing) without employing cross-validation methods. Another possible solution would be, in a future work, to reduce the number of cases inside the highest classes to reduce the uneven data distribution. (4) Additional CVDs should be studied and evaluated in future work to enhance the discriminative capabilities of our algorithm (e.g., arrhythmias such as premature atrial contraction, premature ventricular contraction, and atrial fibrillation).

Nevertheless, upon reviewing state-of-the-art works (refer to Table 1), it becomes apparent that many have encountered similar limitations. These constraints predominantly revolved around imbalances in data distribution, as a significant portion of these studies relied on the same database. Additionally, limitations in computational time and resources, and a restricted variety and diversity of CVD classes were commonly shared among these works. This collective set of limitations across the consulted literature underscores the need for addressing data imbalances and expanding the diversity of CVD classes in future research efforts.

## Figures and Tables

**Table 1:** State-of-the-art literature report on CVDs detection with information about the database, the comparison groups, the features extracted, used classifiers, limitations, and Accuracy.

Ref	Year	Database	Comparison Group (Number of Participants)	Feature Extracted	Classifier	Limitations	Validation	Accuracy
[25]	2004	University Hospital in Lund database	Normal (1119) vs. Myocardial infarction (1119)	Hermite decomposition	ANN	Exclusive assessment of myocardial infarction. Lack of diversity of CVDs.	Cross-validation	94%
[26]	2016	PTB diagnostic ECG database	Normal (49) vs. Cardiomyopathy (14)	ECG PR, QT, RR and QRS intervals	Feed-forward back-propagation Neural Network	Small and unbalanced dataset. Exclusive assessment of cardiomyopathy. Lack of diversity of CVDs.	Cross-validation	95.2%
[27]	2018	PTB diagnostic ECG database	Normal (25) vs. Myocardial infarction (36)	Feature extracted from DNN	DNN (InceptionV3)	Small database for hold-on. Lack of diversity of CVDs.	Hold-on	99.64%
[28]	2018	PTB diagnostic ECG datasets	Normal (52) vs. Myocardial infarction (148)	Features extracted from CNN	CNN	Small and unbalanced database for hold-on. It is impossible to know what features were extracted due to the nature of deep learning algorithms. Lack of diversity of CVDs.	Hold-on	95.9%
[29]	2019	PTB diagnostic ECG database	Healthy (52) vs. Myocardial infarction (148)	Feature extracted from CNN	CNN	Small and unbalanced dataset.There was no discrimination of diseases outside of the MI class. Lack of diversity of CVDs.	Cross-validation	99.78%
[30]	2019	Cleveland heart disease database	Healthy (150) vs. Heart Disease (147)	Patient clinical information	SVM	Small dataset. There was no discrimination of diseases outside of the Heart Disease class. Lack of diversity of CVDs.	Hold-on	92.22%
[31]	2019	Cleveland heart disease database	Healthy (150) vs. Heart Disease (147)	Patient clinical information	DNN	Small dataset. There was no discrimination of diseases outside of the Heart Disease class. Lack of diversity of CVDs.	Hold-on	93.33%
[32]	2020	Heart sound dataset	Normal (400) vs. Mitral valve prolapse (400) vs. Mitral stenosis (400) vs. Mitral regurgitation (400) vs. Aortic stenosis (400)	Feature extracted from CNN	CNN	Low variety of classes. There is a high risk of over-fitting because of the augmentation technique used.	Cross-validation	98.6%
[33]	2020	PTB diagnostic ECG datasets	Normal (313) vs. Abnormal (318)	Features extracted from ANN	Ensemble	Use class weights when training with artificial neural networks to solve the class unbalance problem.	Hold-on	94.14%
[34]	2020	PTB diagnostic ECG database	No Myocardial infarction (141) vs. Myocardial infarction (148)	Feature extracted from CNN	CNN	Small dataset. Just MI different types of discrimination. Small database.	Cross-validation	81%
[35]	2021	Ch. Pervaiz Elahi Institute of Cardiology Multan Dataset	Normal (3408) vs. Abnormal (2796) vs. Myocardial infarction (2880) vs. Previous history of Myocardial infarction (2064)	Feature extracted from SSD MobileNetV2 (CNN)	SSD MobileNetV2 (CNN)	Just MI different types of discrimination. It is impossible to know what features were extracted due to the nature of deep learning algorithms.	Hold-on	98.33%
[36]	2021	PTB diagnostic ECG database	Healthy (52) vs. Abnormal (216)	Domain features and disease-specific features	XGBoost	Small and unbalanced dataset. There is no disease discrimination, just normal vs. abnormal. Lack of diversity of CVDs.	Cross-validation	98.23%
[37]	2021	Cleveland dataset	Normal (170) vs. Abnormal (140)	Patient clinical information	Decision tree and Random forest combined	Small database for hold-on. There is no disease discrimination, just normal vs. abnormal. Lack of diversity of CVDs.	Hold-on	88.00%
[38]	2021	Cleveland HD dataset	Healthy (135) vs. Heart disease (135)	Feature extracted from MAFW	CNN model with the MAFW	Small dataset. A limited number of classifiers were used. High computational cost and time complexity. Runtime is not considered as an evaluation criterion. Lack of diversity of CVDs.	Cross-validation	90.1%
[39]	2022	Cleveland, Hungary, Switzerland, and Long Beach V datasets	Healthy (500) vs. Heart disease (550)	Patient clinical information	Extreme gradient boosting	No disease discrimination, just normal vs. abnormal. Lack of diversity of CVDs.	Cross-validation	100%
[40]	2022	UC Irvine Machine Learning Repository CVD datasets	Healthy (500) vs. Heart disease (550)	Patient clinical information	CNN and BiLSTM hybrid	No disease discrimination, just normal vs. abnormal. Lack of diversity of CVDs.	Hold-on	94.51%
[41]	2022	ECG dataset of Cardiac and COVID-19 Patients and ECG dataset of Cardiac Patients	Normal (284) vs. Abnormal (233) vs. MI (239) vs. Previous history of MI (102)	Feature extracted from MobileNet V2 (CNN)	MobileNet V2 (CNN)	Small database for hold-on, lack of a truly independent test group. Did not consider optimisation techniques.	Hold-on	95.18%
[42]	2022	PTB-XL dataset	Normal (1608) vs. Abnormal (1357)	Feature extracted from DNN	XGBoost	Lack of diversity of CVDs. Unbalanced dataset.	Hold-on	78.65%
Present Work	2023	PTB diagnostic ECG database	VHD vs. *M*, VHD vs. MI; VHD vs. MH; VHD vs. HC; VHD vs. Dis; VHD vs. CardMyo; VHD vs. BBB; *M* vs. MI; *M* vs. MH; *M* vs. HC; *M* vs. Dis; *M* vs. CardMyo; *M* vs. BBB; MI vs. MH; MI vs. HC; MI vs. Dis; MI vs. CardMyo; MI vs. BBB; MH vs. HC; MH vs. Dis; MH vs. CardMyo; MH vs. BBB; HC vs. Dis; HC vs. CardMyo and HC vs. BBB	Approximate Entropy, Logarithmic Entropy, Shannon Entropy, Correlation Dimension, Detrended Fluctuation Analysis, Energy, Higuchi Fractal Dimension, Hurst Exponent, Katz Fractal Dimension and Lyapunov Exponent	19 ML Classifiers	Small data sample for some classes and unbalanced dataset.	Cross-validation	73–100%

**Table 2:** Number of ECGs per diagnosis class.

Diagnostic Class	Number of ECGs
Bundle branch block (BBB)	17
Cardiomyopathy (CardMyo)	20
Healthy controls (HC)	80
Myocarditis (*M*)	4
Myocardial hypertrophy (MH)	4
Myocardial infarction (MI)	367
Valvular heart disease (VHD)	6
Dysrhythmia (Dis)	16

**Table 3:** Number of ECGs per diagnosis class after signal quality assessment and artifacts removal.

Diagnostic Class	Number of ECGs
Bundle branch block	9
Cardiomyopathy	15
Healthy controls	75
Myocarditis	3
Myocardial hypertrophy	4
Myocardial infarction	362
Valvular heart disease	4
Dysrhythmia	11

**Table 4:** The extracted features with the corresponding equations and definitions.

Feature	Equation	Definition
Approximate Entropy (ApEn)	$ApEn\left(m,r\right)=\lim_{N\to \infty }\Theta^{m}\left(r\right)-\Theta^{m+1}\left(r\right),$ Θ is the Heaviside step function and *m* is the dimension [52].	ApEn evaluates the likelihood that similar patterns within the data will remain similar when additional data points are included. The lower the ApEn value is, the more regular or predictable the data are, whereas a higher ApEn value suggests greater complexity or irregularity.
Correlation Dimension (CorrDim)	$$CorrDim=\lim_{M\to \infty }\frac{2\sum_{i=1}^{M-k}\sum_{j=i+k}^{M}\Theta \left(l|X_{i}-X_{j}|\right)}{M^{2}},$$ where Θ(x) is the Heaviside step function, $X_{i}$ and $X_{j}$ are the position vectors on attractor, *l* is the distance under consideration, *k* is the summation offset, and *M* is the reconstructed vector numbers from the x(n) [52].	CorrDim is used to measure self-similarity, and higher values of CorrDim means a high degree of complexity and less similarity.
Detrended Fluctuation Analysis (DFA)	$$DFA\left(n\right)=\sqrt{\frac{\sum_{k=1}^{N}{\left[y\left(k\right)-y_{n}\left(k\right)\right]}^{2}}{N}},$$ where *N* is the length, $y_{n}\left(k\right)$ is the local trend, and y(k) is defined as $$y\left(k\right)=\sum_{i=1}^{k}\left[x\left(i\right)-\bar{x}\right],$$ with x(i) as the inter-beat interval and $\bar{x}$ as its average [53].	DFA is a technique for measuring the power scaling observed through R/S analysis.
Energy (En)	$En=\sum_{n=0}^{N-1}|x\left(n\right){|}^{2}$	En is the capacity of a system to perform work [54].
Higuchi Fractal Dimension (*H*)	$H=\frac{\ln(L(k))}{\ln(\frac{1}{k})},$ where *k* is a number of composed sub-series and L(k) is the averaged curve size.	*H* estimates the fractal dimension of a time series signal [55].
Hurst Exponent (EH)	$K_{q}\left(\tau \right)∼{\left(\frac{\tau }{\nu }\right)}^{qEH(q)},$ with $K_{q}\left(\tau \right)=\frac{\left(|X\left(t+\tau \right)-X\left(t\right)\right){|}^{q})}{\left(|X\left(t\right){|}^{q}\right)},$ where *q* is the order moments of the distribution increments, ν is the time resolution, τ is the incorporation time delay of the attractor, and *t* is the period of a given time series signal X(t) [56].	EH quantifies how chaotic or unpredictable a time series is.
Katz Fractal Dimension (*K*)	$K=\frac{\log(n)}{\log\left(n\right)+\log\left(\frac{max_{n}\left(\sqrt{{\left(n\;-\;1\right)}^{2}\;+\;{\left(x\left(n\right)\;-\;x\left(1\right)\right)}^{2}}\right)}{\sum_{n=2}^{N}\sqrt{1\;+\;{\left(x\left(n\;-\;1\right)\;-\;x\left(n\right)\right)}^{2}}}\right)},$	*K* estimates the fractal dimensions through a waveform analysis of a time series [56].
Logarithmic Entropy (LogEn)	$LogEn=\sum_{n=1}^{N}\log_{2}\left[|x\left(n\right){|}^{2}\right]$	LogEn quantifies the average amount of information (in bits) needed to represent each event in the probability distribution. Higher logarithm entropy values indicate greater unpredictability or randomness in the distribution, while lower values suggest more certainty or order [54].
Lyapunov Exponent (Elay)	$$ELay\left(x_{0}\right)=\lim_{n\to \infty }\frac{\sum_{k=1}^{n}\ln|f^{\prime }\left(x_{k}-1\right)|}{n},$$ where $f^{\prime }$ is the *f* derivative [57].	ELya evaluates the system’s predictability and sensitivity to change.
Shannon Entropy (ShaEn)	$ShaEn=-\sum_{n=1}^{N}|x\left(n\right){|}^{2}\log_{2}\left[|x\left(n\right){|}^{2}\right]$	ShaEn is measured in bits when the base-2 logarithm (log2) is used. This means that the result provides a quantification of the average number of bits required to represent each outcome in a given probability distribution. Higher entropy values indicate greater uncertainty, unpredictability, or randomness in the distribution, while lower values suggest more order or certainty [54].

**Table 5:** 19 Sci-learn ML classifiers configurations.

Classifier	Hyperparameters
AdaBoostClassifier (AdaBoost)	Default parameters
BaggingClassifier (BaggC)	Default parameters
DecisionTreeClassifier (DeTreeC)	max_depth: 5
ExtraTreesClassifier (ExTreeC)	n_estimators: 300
GaussianNB (GauNB)	Default parameters
GaussianProcessClassifier (GauPro)	1.0 × RBF(1.0)
GradientBoostingClassifier (GradBoost)	Default parameters
KNearestNeighborsClassifier (KNN)	Default parameters
LinearDiscriminantAnalysis (LinDis)	Default parameters
LinearSVC (LinSVC)	Default parameters
LogisticRegression (LogReg)	solver: “lbfgs”
LogisticRegressionCV (LogRegCV)	cv: 3
MLPClassifier (MLP)	α: 1, max_iter: 1000
OneVsRestClassifier (OvsR)	random_state: 0
RandomForestClassifier (RF)	max_depth: 5, n_estimators: 300, max_features: 1
SGDClassifier (SGD)	max_iter: 100, tol: 0.001
SGDClassifierMod (SGDCMod)	Default parameters
Support-vector Machines (SVC)	γ: “auto”

**Table 6:** Statistical and XROC results for individual feature power analysis per binary groups, where N.S. means no significance.

Comparison Group	Feature	Compressor	*m*th Sub-Band	Lead	*p*-Value	Recall	Accuracy
VHDvs.M	CorrDim	Avg	2	V4	0.0339	100%	100%
VHDvs.MI	CorrDim	Avg	2	*I*	0.0486	0%	98.91%
VHDvs.MH	CorrDim	Avg	2	V1	N.S.	75.00%	87.50%
VHDvs.HC	CorrDim	Avg	2	Vy	0.0301	0%	94.94%
VHDvs.Dis	CorrDim	Avg	2	V5	N.S.	100%	80.00%
VHDvs.CardMyo	CorrDim	Avg	2	Vy	N.S.	50.00%	78.94%
VHDvs.BBB	CorrDim	Avg	2	V1	0.0308	75.00%	84.62%
Mvs.MI	CorrDim	Avg	2	V4	N.S.	0%	99.18%
Mvs.MH	CorrDim	Avg	2	III	N.S.	100%	85.71%
Mvs.HC	CorrDim	Avg	2	V6	N.S.	0%	96.15%
Mvs.Dis	CorrDim	Avg	2	V2	N.S.	33.33%	85.71%
Mvs.CardMyo	CorrDim	Avg	2	V2	N.S.	66.67%	94.44%
Mvs.BBB	CorrDim	Avg	2	V4	0.0126	100%	100%
MIvs.MH	CorrDim	Avg	2	II	N.S.	100%	98.91%
MIvs.HC	CorrDim	Avg	2	V6	0.0017	100%	82.84%
MIvs.Dis	CorrDim	Avg	2	Vy	N.S.	100%	97.05%
MIvs.CardMyo	CorrDim	Avg	2	V6	0.0326	100%	96.02%
MIvs.BBB	CorrDim	Avg	2	aVr	0.0027	100%	97.57%
MHvs.HC	CorrDim	Avg	2	II	0.0078	0%	94.94%
MHvs.Dis	CorrDim	Avg	2	Vz	N.S.	50.00%	80.00%
MHvs.CardMyo	CorrDim	Avg	2	Vz	N.S.	50.00%	84.21%
MHvs.BBB	CorrDim	Avg	2	V2	N.S.	75.00%	92.31%
HCvs.Dis	CorrDim	Avg	2	V1	N.S.	100%	87.21%
HCvs.CardMyo	CorrDim	Avg	2	III	0.0071	94.67%	83.33%
HCvs.BBB	CorrDim	Avg	2	V2	0.0047	100%	89.29%
Disvs.CardMyo	CorrDim	Avg	2	V3	N.S.	54.54%	73.08%
Disvs.BBB	CorrDim	Avg	2	aVr	0.0167	81.81%	80.00%
CardMyovs.BBB	CorrDim	Avg	2	aVr	N.S.	73.33%	75.00%

**Table 7:** The total number of occasions that a feature was shown to be statistically significant (p<0.05) across all sub-band analyses and leads.

Comparison Group	ApEn	CorrDim	DFA	En	*H*	EH	*K*	LogEn	Elay	ShaEn	Total
VHDvs.M	48	42	48	48	48	48	48	48	48	48	474
VHDvs.MI	24	21	24	24	24	24	24	24	24	24	237
VHDvs.MH	0	0	0	0	0	0	0	0	0	0	0
VHDvs.HC	48	42	48	48	48	48	48	48	48	48	474
VHDvs.Dis	0	0	0	0	0	0	0	0	0	0	0
VHDvs.CardMyo	0	0	0	0	0	0	0	0	0	0	0
VHDvs.BBB	24	21	24	24	24	24	24	24	24	24	237
Mvs.MI	0	0	0	0	0	0	0	0	0	0	0
Mvs.MH	0	0	0	0	0	0	0	0	0	0	0
Mvs.HC	0	0	0	0	0	0	0	0	0	0	0
Mvs.Dis	0	0	0	0	0	0	0	0	0	0	0
Mvs.CardMyo	0	0	0	0	0	0	0	0	0	0	0
Mvs.BBB	24	21	24	24	24	24	24	24	24	24	237
MIvs.MH	0	0	0	0	0	0	0	0	0	0	0
MIvs.HC	120	105	120	120	120	120	120	120	120	120	1185
MIvs.Dis	0	0	0	0	0	0	0	0	0	0	0
MIvs.CardMyo	48	42	48	48	48	48	48	48	48	48	474
MIvs.BBB	48	41	48	48	48	48	48	48	48	48	473
MHvs.HC	24	21	24	24	24	24	24	24	24	24	237
MHvs.Dis	0	0	0	0	0	0	0	0	0	0	0
MHvs.CardMyo	0	0	0	0	0	0	0	0	0	0	0
MHvs.BBB	0	0	0	0	0	0	0	0	0	0	0
HCvs.Dis	0	0	0	0	0	0	0	0	0	0	0
HCvs.CardMyo	48	42	48	48	48	48	48	48	48	48	474
HCvs.BBB	120	104	120	120	120	120	120	120	120	120	1184
Disvs.CardMyo	0	0	0	0	0	0	0	0	0	0	0
Disvs.BBB	24	21	24	24	24	24	24	24	24	24	237
CardMyovs.BBB	0	0	0	0	0	0	0	0	0	0	0
Total	600	523	600	600	600	600	600	600	600	600	

## Data Availability

The data presented in this study are available on request from the corresponding author (accurately indicate status).
